# How does the extent of fibrosis in adenomyosis lesions contribute to heavy menstrual bleeding?

**DOI:** 10.1002/rmb2.12442

**Published:** 2022-02-07

**Authors:** Qingqing Huang, Xishi Liu, Hilary Critchley, Zhongpeng Fu, Sun‐Wei Guo

**Affiliations:** ^1^ Department of Gynecology The Third Affiliated Hospital of Guangzhou Medical University Guangzhou Guangdong China; ^2^ Department of Gynecology Shanghai OB/GYN Hospital Fudan University Shanghai China; ^3^ Shanghai Key Laboratory of Female Reproductive Endocrine‐Related Diseases Fudan University Shanghai China; ^4^ MRC Centre for Reproductive Health University of Edinburgh The Queen's Medical Research Institute Edinburgh UK; ^5^ Department of Ultrasound Imaging Shanghai OB/GYN Hospital Fudan University Shanghai China; ^6^ Research Institute Shanghai OB/GYN Hospital Fudan University Shanghai China

**Keywords:** adenomyosis, fibrosis, heavy menstrual bleeding, hypoxia, prostaglandin E2

## Abstract

**Purpose:**

To investigate how the extent of fibrosis in adenomyosis lesions contributes to heavy menstrual bleeding (HMB).

**Methods:**

We recruited 57 women with histologically confirmed adenomyosis, 29 of whom reported moderate/heavy bleeding (MHB) (menstrual blood loss (MBL) ≥20 but <100 mL) and the remaining 28, excessive MBL (EXB; ≥100 mL). Lesional stiffness was measured by transvaginal elastosonography. Full‐thickness uterine tissue columns containing the lesion and its neighboring endometrial‐myometrial interface (EMI) and endometrial tissues were evaluated for tissue fibrosis and immunohistochemical analysis of HIF‐1α, COX‐2, EP2, and EP4.

**Results:**

The lesional stiffness in the EXB group was significantly higher than that of MHB, and consistently, the extent of lesional fibrosis and the extent of tissue fibrosis in both EMI and eutopic endometrium were also significantly higher. In adenomyotic lesions and their neighboring EMI and eutopic endometrial tissues, the immunostaining of HIF‐1α, COX‐2, EP2, and EP4 was significantly reduced. The extent of fibrosis and the immunostaining levels of HIF‐1α, COX‐2, EP2, and EP4 were negatively correlated in all tissues.

**Conclusions:**

Lesional fibrosis begets stiffening matrix, propagating fibrosis to neighboring EMI and eutopic endometrium, resulting in reduced PGE_2_ and HIF‐1α signaling, and thus likely reduced hypoxia necessary for endometrial repair, leading to HMB.

## Graphical abstract

We provide the first piece of evidence that the extent of fibrosis in adenomyotic lesions can propagate to its neighboring endometrial‐myometrial interface and endometrium, reducing the hypoxia and PGE2 signaling and causing impaired endometrial repair, and consequently, heavy menstrual bleeding.

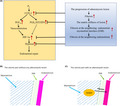

## 1 INTRODUCTION

Heavy menstrual bleeding (HMB) is defined in contemporary times as excessive menstrual blood loss (MBL) that disrupts physical, social, emotional, and/or material quality of life (NICE Guideline, 2018). Objective measurement of MBL defines HMB as MBL greater than 80 mL.^1^, ^2^ HMB affects as many as one in three women over their reproductive age span and is one of most prevalent gynecological disorders for which women seek medical attention,^3^ and is yet still under reported.^4^ The debilitating symptom of HMB is associated with significantly reduced quality of life^5^ and exerts a heavy economic burden on the afflicted women, their family, and society.^6^ While medical treatments for HMB are available, they are mostly hormonal and often have limited efficacy or troublesome side effects,^7^ which are attributable, perhaps in no small part, to the fragmented understanding of the molecular and cellular mechanisms underlying HMB.

The classification acronym for abnormal uterine bleeding (AUB) in the reproductive years, which includes the symptom of HMB, “PALM‐COEIN”,^8^ identifies adenomyosis as one important contributing factor to HMB (AUB‐A). However, the mechanisms underlying adenomyosis‐induced HMB are poorly understood,^9^ and, as a result, hysterectomy is often the definitive solution.

In the last few years, burgeoning evidence has outlined the natural history of adenomyosis. In essence, adenomyotic lesions, defined to be the adenomyotic foci within the myometrium, are fundamentally “wounds” undergoing repeated tissue injury and repair (ReTIAR) like their endometriotic counterpart, and through epithelial–mesenchymal transition (EMT), fibroblast‐to‐myofibroblast transdifferentiation (FMT), and smooth muscle metaplasia (SMM), the lesions progressively become more fibrotic.^10^, ^11^, ^12^, ^13^ Capitalizing on this reported progressive fibrogenesis and ensuing tissue stiffening, as well as on the capability of elastography, a new non‐invasive imaging technique to measure tissue stiffness, in detecting the extent of fibrosis,^14^ ultrasound elastography can be utilized to aid diagnosis of adenomyosis.^15^ However, it is unclear how insights into the natural history of adenomyotic lesions will help us better understand the pathophysiology of the disease, such as adenomyosis‐related HMB.

Following the withdrawal of progesterone in the late secretory phase in the absence of pregnancy, a pivotal event for menstrual induction, endometrial expression of cyclooxygenase‐2 (COX‐2), a gene coding for the rate‐limiting prostanoid synthetic enzyme, is elevated, resulting in subsequent increased levels of prostaglandins (PGs), namely, PGE_2_ and PGF_2α_.^16^, ^17^ Increased endometrial PGE_2_ signaling, along with endometrial generation of the vasoconstrictor, PGF_2α_, leads to local hypoxia in the upper functional layer of the endometrium. This hallmark event of activation of hypoxia inducible factor 1α (HIF‐1α), the master regulator of cellular hypoxia, creates an endometrial microenvironment that is conducive to tissue repair and angiogenesis.^18^, ^19^, ^20^ Disruption of the hypoxia signaling or the PGE_2_ signaling in endometrium would impair endometrial repair, causing HMB.^9^, ^20^

In fibrotic conditions elsewhere in the body, for example, in pulmonary fibrosis, it is reported that PGE_2_ is anti‐fibrotic.^21^, ^22^, ^23^, ^24^ With the progression of fibrogenesis, and in the present context of uterine adenomyosis,^10^, ^11^, ^25^, ^26^ the lesional stiffness has been observed to increase,^15^ which thus would be accompanied by downregulation of COX‐2 expression and consequent decrease in PGE_2_ production.^27^ Indeed, we recently reported that, in the context of endometriosis, the expression of COX‐2 and E‐series receptor type 2 (EP2) and EP4 is reduced as endometriotic lesions became more fibrotic.^28^, ^29^ In addition, endometriotic stromal cells cultured in stiffer matrices demonstrate decreasing expression of COX‐2, EP2, and EP4,^28^ suggesting reduced PGE_2_ signaling as the extracellular matrix (ECM) becomes stiffer. As ectopic endometrium, both adenomyosis and endometriotic lesions share the same and defining hallmark of cyclic bleeding,^30^ they also share similar, but not exactly identical molecular processes that underpin lesional progression owing to different microenvironment.^13^ These lines of evidence raise the possibility that, as adenomyotic lesions become progressively fibrotic and thus increasingly stiffened, the behavior of neighboring eutopic endometrial cells may be modified. This modification is highly likely in light of a recent report that adenomyosis lesions are stereoscopically characterized by an “ant colony‐like network” that directly connects with endometrial glands.^31^ This may lead to modulation of local PG signaling with reduced expression of COX‐2, EP2, and EP4, and thus reduced PGE_2_, which, in turn, may result in suppression of HIF‐1α and subsequent impaired endometrial repair and, finally, HMB. It also may form a “patchy” and heterogeneous endometrium with different patches exhibiting various, disparate, and even dissimilar molecular signatures.

Therefore, we hypothesized that women with adenomyosis who complain of HMB have a greater extent of lesional fibrosis and thus greater stiffness, as well as reduced PGE_2_ signaling, leading to reduced hypoxia signaling, which would be associated with impaired endometrial repair. This would manifest as reduced immunostaining of HIF‐1α, COX‐2, EP2, and EP4 in adenomyotic lesions and their neighboring endometrial–myometrial interface (EMI) and eutopic endometrium. To test this hypothesis, we employed ultrasound elastography to assess lesional stiffness in women with adenomyosis who complained of moderate–heavy (MHB) and excessive menstrual bleeding (EXB). We further performed immunohistochemical localization of HIF‐1α, COX‐2, EP2, and EP4, as well as quantification of tissue fibrosis through Masson trichrome staining, in uterine adenomyotic lesions, and their neighboring EMI and endometrial tissues.

## 2 MATERIALS AND METHODS

### 2.1 Ethics statement

The design, analysis, interpretation of data, manuscript drafting, and revisions conform with the Helsinki Declaration, the Committee on Publication Ethics (COPE) guidelines (http://publicationethics.org/), and the RECORD (REporting of studies Conducted using Observational Routinely‐collected health Data) statement, available through the EQUATOR (enhancing the quality and transparency of health research) network (www.equator‐network.org). The study was approved by the institutional ethics review board of Shanghai OB/GYN Hospital (Date of approval: March 6, 2019; on file), Fudan University, China. Each patient enrolled in this study signed an informed consent for all the procedures and to allow data collection and analysis for research purposes. The study was non‐advertised, and no remuneration was offered to encourage patients to give consent.

### 2.2 Patient demographics

**FIGURE 1:**
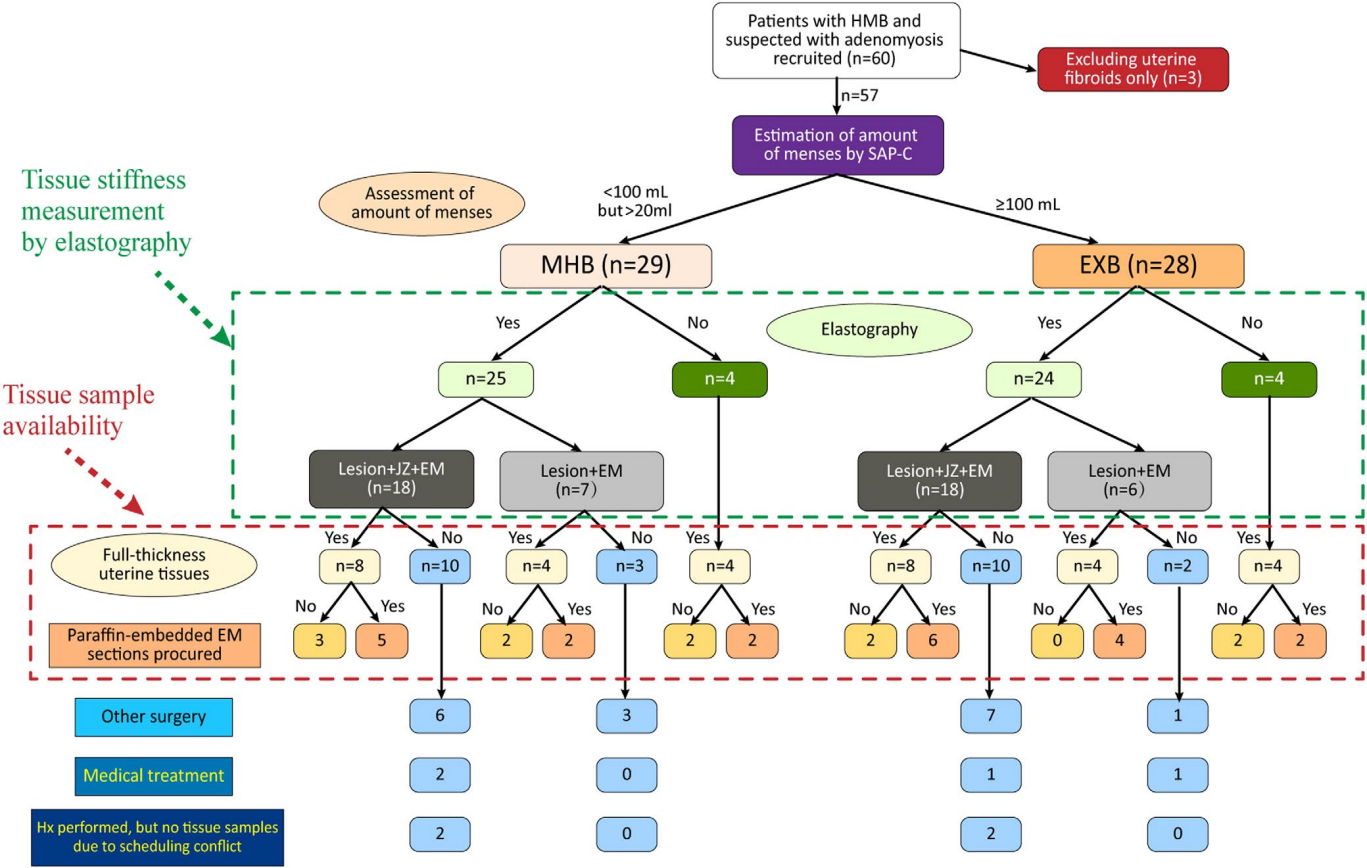
Flow chart demonstrating patient recruitment, grouping, tissue stiffness by transvaginal elastosonography (TVESG), and tissue availability for analyses. Abbreviations used: EXB: excessive menstrual bleeding; HMB: heavy menstrual bleeding; Hx: Hysterectomy; MHB: moderate–heavy menstrual bleeding

Sixty premenopausal patients, who visited Shanghai OB & GYN Hospital, Fudan University, from July to December, 2019, were recruited to this study. Participants were initially diagnosed with adenomyosis based on a combination of transvaginal ultrasound (TVUS) findings, symptoms of HMB, and gynecological examination, and for whom laparoscopic hysterectomy or medical treatment was advised. These patients were all free of any hematological disorders at their initial screening, and none of the recruited patients had taken any anti‐platelet, hormonal preparation, oral contraceptive, anti‐diabetic, or other medications at least 3 months prior to their participation in this study. Three participants were later excluded because of the finding of uterine fibroids but no adenomyosis at the time of hysterectomy/confirmed by histology, leaving 57 subjects with histologically confirmed adenomyosis. A flow chart demonstrating patient recruitment, grouping, measurement of tissue stiffness by TVESG, and availability of tissue samples for analyses is presented in Figure 1.

For all recruited patients, their medical records, including clinical symptoms, features, and pathological reports, were carefully reviewed and their data retrieved. The demographic and clinical information on age, gravidity, parity, menstrual cycle phase, length of menstrual cycles, date of reported first day of last menstruation, verbal descriptor scale (VDS, i.e., none, mild, moderate, or severe) on symptom of dysmenorrhea, the amount of MBL (as described below), hemoglobin levels (in g/L), use of iron supplementation, uterine size as measured by ultrasound, the date of surgery, and the past medical history (including, surgical history) were collected. For all patients, diffuse or focal adenomyosis was distinguished by pathological reports of uterine histology for patients who underwent hysterectomy.

### 2.3 Clinical assessments

#### 2.3.1 MBL estimation method

Of the 57 recruited patients, 29 reported moderate or heavy menses (the moderate‐to‐heavy bleeding group, or MHB) based on their MBL (<80 mL but >20 mL, and ≥80 but <100 mL, respectively) as estimated by the menstrual pictogram (SAP‐c version),^32^ and the remaining 28 complained of excessive MBL (the excessive bleeding group, or EXB, defined to be ≥100 mL). Patients in both groups were further evaluated with TVESG examination, except 4 each in the MHB and EXB groups (13.8% and 14.3%, respectively) for whom TVESG examination was not performed due to conflict with the scheduled surgery time.

As described in,^32^ the menstrual pictogram (SAP‐c version) includes three Always Ultra towel types (widely available in China): normal, long, and night, each of which comprises five diagrams (icons) depicting different dimensions and absorbency ratings. Each icon was assigned an actual blood volume that was validated against the alkaline hematin method.^1^, ^33^, ^34^ Patients were instructed to compare each soiled menstrual product, used during their menstrual cycle, to the relevant set of icons on the menstrual pictogram and decide which image it most closely resembled. The total MBL amount was arrived by converting the scores of the menstrual pictogram (SAP‐c version). In addition, each patient's hemoglobin level (in g/L) prior to the surgery/treatment was recorded.

#### 2.3.2 TVUS and TVESG

Once recruited to the study, all patients were invited to receive further evaluation with TVUS (B‐mode) and TVESG before surgery to measure tissue stiffness, as described below, except 4 each in the MHB and EXB groups (13.8% and 14.3%, respectively) could not undergo the TVESG examination due to the conflict with the scheduled operation time (Figure 1).

For patients who had undergone TVESG, the stiffness of their adenomyotic lesions was measured and the region (of interest, or ROI) with the highest stiffness was recorded and designated for tissue sampling. In addition, the stiffness of the eutopic endometrium, as well as the EMI later on, proximal to the designated ROI in the lesion was also measured and recorded. These three locations were determined by the conventional TVUS (B‐mode) and TVESG, as described below. There were 18 patients each in the MHB and EXB groups (62.1% and 64.3%, respectively) with the stiffness of adenomyotic lesions and their neighboring EMI and eutopic endometrium measured by TVUS (B‐mode) and TVESG, and there were 7 (24.1%) and 6 (21.4%) in the MHB and EXB groups, respectively, with the stiffness of adenomyosis lesions and eutopic endometrium, but not of EMI, measured by TVESG (which were cases recruited at the very beginning of the study).

For all recruited subjects, TVUS and TVESG were both performed on a Hitachi Aloka ARIETTA 70 with an EUP‐V53W transvaginal probe (Hitachi, Tokyo, Japan) and by the same sonographer (ZF), who had over 10 years of experience in obstetric and gynecologic ultrasound and had received advanced training in operating TVESG. This ultrasound system is a real‐time strain elastography that detects tissue strain while compressing the surface with the transducer. During acquisition of an elastographic image, the machine quantifies the tissue displacement by tracking ultrasonographic speckles and comparing changes in displacement (which reflects tissue stiffness) before and after pressure application (excitation). The amount of change in deformation, as a measure of stiffness, is color coded and is superimposed on the corresponding B‐mode image, with red being the softest and blue being the stiffest, and green being the average stiffness. Therefore, the eutopic endometrium and serosa mainly showed as red, while the adenomyotic lesions showed as primarily blue. The color of the EMI, of which thickness is at least 8–12 mm in adenomyosis,^35^ depended on its stiffness.

All recruited patients were asked to empty their bladder before examination and were placed in the lithotomy position. TVUS (B‐mode) was first performed to evaluate the uterus, the myometrium, and the endometrium (including uterine size, location, type, extent, and size of the adenomyotic lesions). For diagnosing adenomyosis by TVUS, the diagnosis was made based on features described by Ferraz et al.,^36^ which, in turn, is based on the Morphological Uterus Sonographic Assessment (MUSA) criteria.^37^ This was followed by the TVESG to obtain images by using compression, with the probe that lightly pressed against the location of interest, causing little deformation. The manual‐controlled force exerted by the probe was maintained as consistent as possible for each and every patient. The sonographer varied the pressure by making intermittent movements with the probe. Application of a small amount of pressure was followed by a release state with less pressure, with a frequency of approximately one to two times per second. To eliminate possible bias and to maintain consistency, a probe pressure that maintained the strain indicator strictly in a narrow range of 3 to 4 (in 1–8 scale) was used for all patients. The strain indicator showed whether enough tissue displacement was achieved to adequately calculate local strains within the region of interest (ROI) and was displayed in real time as a feedback to the examiner. The typical lesional area of the uterus was first defined by TVUS (B‐mode) and then TVESG, which showed the dark blue area (the stiffest), as the target locations of the uterus. Once the high‐quality and reproducible image was obtained, the ROI was set at the target location. Then, the stiffness at the ROI of the uterus, including adenomyotic lesion and its neighboring EMI and eutopic endometrium, was measured. In addition, all subjects were evaluated by one single sonographer (ZF), who was also intentionally kept blinded to the patient group allocation during the entire period of this study.

We include the following as important background to the measurement technique employed. It should be noted that the Hitachi machine used in this study did not provide an absolute reading for tissue stiffness. Instead, it provides a so‐called *liver function index* or LFI, which is a built‐in stiffness measurement implemented in the Hitachi machines and is displayed automatically after the ROI is positioned. LFI is based on regression of 11 co‐variables derived from machine readings, such of kurtosis, degree of strain, and several quantitative characteristics that indicate the contrast, homogeneity, complexity, uniformity, and direction of tissue texture. The regression equation, initially based on studies by Tatsumi et al.^38^, ^39^ and later improved by Fujimoto et al.,^40^ was derived to measure the extent of *liver fibrosis* and to correlate as much as possible with the *liver function per se*.

**FIGURE 2:**
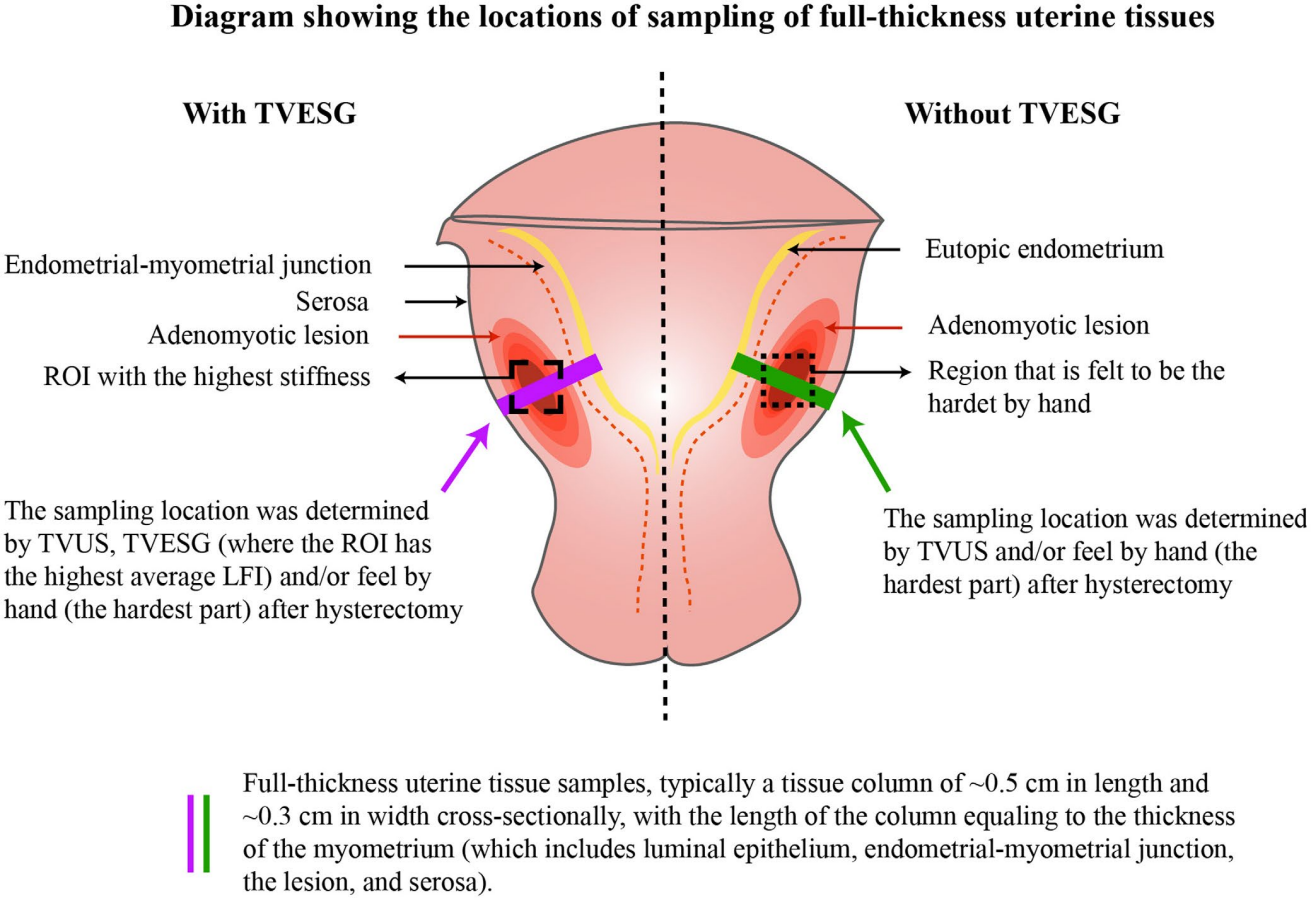
Schematic diagram showing the locations of the sampling of full‐thickness uterine tissue columns for patients who had or had not received TVESG evaluation. Note that the locations of adenomyotic lesions are for demonstration only, since the actual locations of lesions may vary from patient to patient

In all cases, the site of the typical lesion in the uterus (such as in the anterior, or posterior uterine wall, or in the uterine fundus) was also recorded, which was designated to procure the full‐thickness tissue column. Figure 2 depicts how the full‐thickness uterine tissue columns were collected in cases when lesional stiffness measurement was and was not available.

#### 2.3.3 Pathological assessments of uterus

We have reported that the stiffness of adenomyotic lesions is higher than that of leiomyoma, which, in turn, is stiffer than normal myometrium.^15^ In addition, in contrast to leiomyoma lesions, which have pseudo capsules essentially serving as a demarcation with myometrium, there is no similar demarcation between adenomyosis lesions and their surrounding myometrium. Therefore, we first located the firmest part that matched the ultrasound image of the adenomyotic by palpation of the uterus removed in hysterectomy and then procured the uterine tissue column containing the stiffest lesions. In addition, we confirmed our tissue authenticity of adenomyotic lesions under the microscope. Full‐thickness uterine tissues columns (~0.5 cm in length and ~0.3 cm in cross‐sectional width, with the length of the column equal to the thickness of the uterine tissue) containing the adenomyotic lesions, the EMI and eutopic endometrium, were procured from 16 patients each from MHB and EXB groups (55.2% and 57.1%, respectively), who underwent hysterectomy, and their adenomyosis was confirmed by histology. Among them, there were 12 women, each in the MHB and EXB groups (41.4% and 42.9%, respectively) following TVUS (B‐mode) and TVESG examinations, including women with the stiffness of uterine adenomyotic lesions, eutopic endometrium, and the EMI (*n* = 8, each groups) and those with the stiffness of uterine adenomyotic lesions and eutopic endometrium (*n* = 4, each groups). In both the MHB and EXB groups, 4 women, in each group, underwent hysterectomy but did not undergo TVESG assessment (as mentioned above).

The stiffness of uterine adenomyotic lesions, the eutopic endometrium, and the EMI were measured and recorded in patients who had undergone TVUS (B‐mode) and TVESG. We collected uterine adenomyotic lesions from the area where the tissue stiffness had been determined by TVESG, and the neighboring eutopic endometrium and EMI tissue samples to ensure that the site of harvested tissues was consistent with the area that had a highest average stiffness reading by TVESG assessment. Although 2 uterine tissues in the MHB and 3 in the EXB could not be procured in whole after hysterectomy, we still collected the three tissue samples based on the result of TVUS and TVESG and the firmest place palpated by hand after hysterectomy. As for those participants who did not undergo TVESG assessment, we collected their tissue samples based on the result of conventional TVUS and the firmest place palpated by hand after hysterectomy.

Tissue samples were not available from 13 (44.8%) and 12 (42.9%) patients from the MHB and EXB groups, respectively, due to either myomectomy (9 and 8 patients, respectively), or choice of medical treatment (rather than hysterectomy: 2 each from the two groups), or due to scheduling conflict (2 each from the two groups; Figure 1).

In all patients, the co‐occurrence of ovarian endometrioma, deep endometriosis, or uterine fibroids, if any, was determined by laparoscopy and subsequent histology.

### 2.4 Cellular assessments

#### 2.4.1 Immunohistochemical (IHC) analyses

Paraffin‐embedded tissue blocks were sent to the Shanghai branch of Servicebio (Wuhan, China) for sectioning. Initially, endometrial tissue samples from 7 and 4 patients from the MHB and EXB groups, respectively, were not obtained due to circumstances out of our control. Serial 4‐mm sections were obtained from each histological block, with the first resultant slide stained for hematoxylin and eosin (H&E) to confirm the pathological diagnosis, and IHC staining for hypoxia inducing factor 1α (HIF‐1α), COX‐2, E‐series of prostaglandin receptors Type 2 (EP2), and EP4 was performed for full‐thickness uterine tissue samples, including uterine adenomyosis lesions, eutopic endometrium, and the EMI. Routine deparaffinization and rehydration procedures were performed as described previously.^41^

For antigen retrieval, the slides were heated at 98°C in a citric acid buffer (pH 6.0) for a total of 30 minutes and cooled naturally to room temperature. Sections were then incubated with the primary antibody against HIF‐1α (1:100; Abcam, Cambridge, England), COX‐2 (1:100; Abcam), EP2 (1:500; Abcam), or EP4 (1:100; Bioss, Beijing, China) overnight at 4 °C. After slides were rinsed, the horse radish peroxidase (HRP)‐labeled secondary anti‐rabbit/mouse antibody detection reagent (Shanghai Sun BioTech Company, Shanghai) was incubated at room temperature for 30 minutes. The bound antibody complexes were stained for about 1–2 minutes or until appropriate for microscopic examination with diaminobenzidine and then counterstained with hematoxylin (30 seconds) and mounted. To ensure consistency, accuracy, and reliability, a series of 3 to 5 randomly selected images on several sections were taken to obtain a mean value for each immunohistochemistry measure/protein.

More details on IHC procedures and Masson trichrome staining are provided in the Supplementary Information. The representative photomicrographs for negative and positive staining controls are shown in Figure S1.

### 2.5 Statistical analysis

The comparison of distributions of continuous variables between or among two or more groups was made using the Wilcoxon and Kruskal tests, respectively. Pearson's or Spearman's rank correlation coefficient was used when evaluating correlations between two variables when both variables were continuous or when at least one variable was ordinal. To evaluate which factors were associated with the tissue stiffness, multiple linear regression analysis was used. To determine which co‐variables were associated with excessive (as opposed to moderate‐to‐heavy) menses, a multiple logistic regression analysis was used. *P* values of <0.05 were considered statistically significant. All computations were made with R version 4.0.4.^42^

## 3 RESULTS

**FIGURE 3:**
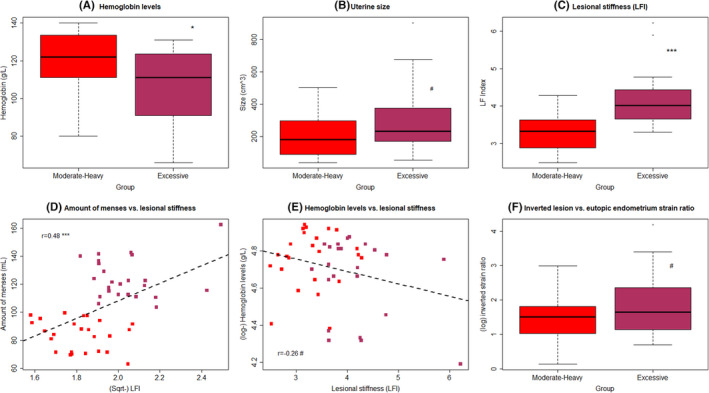
Box plots of the hemoglobin levels (A), uterine size (B), and lesional stiffness, as measured by TVESG (C), in patients with adenomyosis who reported symptoms of moderate‐heavy MBL (MHB) and excessive MBL (EXB). (D) Scatter plots of amount of MBL versus lesional stiffness in patients with moderate–heavy MBL (MHB, red‐colored dots) and excessive MBL (EXB, purple‐colored dots). The dashed line represents the regression line. The number shown is the correlation coefficient, along with the symbol showing the levels of statistical significance. (E) Scatter plots of hemoglobin levels versus lesional stiffness patients in MHB (red‐colored dots) and EXB (purple‐colored dots) groups. The dashed line represents the regression line. The number shown is the correlation coefficient, along with the symbol showing the levels of statistical significance. (F) Boxplot of the inverted strain ratio of lesions vs. eutopic endometrium between MHB and EXB groups. In boxplots, the upper and lower sides of the box represents the upper and lower quartiles, respectively, while the two whiskers represent the minimal and maximal values. The data points outside of the whiskers are outliers. The definitions for MHB and EXB are given in the text and Figure 1. Symbols for statistical significance levels: #: 0.05 < *p *< 0.09; *: *p *< 0.05; **: *p *< 0.01; ***: *p *< 0.001; #: *p* > 0.05. TVESG indicates transvaginal elastosonography

The characteristics of patients recruited for this study are described in Table 1. From Table 1, we observed that, as expected, patients in the EXB group had significantly greater MBL and lower Hb levels than those patients in the MHB group (Figure 3A), as the amount of menses correlated negatively with the Hb levels (*r* = −0.62, *p *= 4.8 × 10^−7^). The two groups were comparable, and the patients in the EXB group had slightly larger uterine sizes, but the difference did not reach statistically significance (*p *= 0.089; Figure 3B). Multiple regression analysis incorporating age, parity, menstrual cycle phase, type of adenomyosis, iron supplementation or not, co‐occurrence of ovarian endometrioma, deep endometriosis, or uterine fibroids and group identity (MHB vs. EXB) indicated that age was positively (*p *= 0.0067) associated, while the co‐occurrence of endometrioma and the secretory phase was negatively associated (*p *= 0.0002, and *p *= 0.0012) with the uterine size (*R^2^* = 0.46).

**TABLE 1:** Characteristics of patients with adenomyosis and symptoms of moderate–heavy menstrual bleeding and excessive menstrual bleeding

Variable	Moderate‐Heavy bleeding (*n* = 29)	Excessive bleeding (*n* = 28)	*P* value
Age (in years)			
Mean ± SD	42.6 ± 6.6	43.1 ± 5.1	0.75
Median (range)	45 (24–48)	44 (32–49)	
Menstrual phase			
Proliferative	17 (58.6%)	21 (75.0%)	0.26
Secretory	12 (41.4%)	7 (25.0%)	
Parity			
0	4 (14.3%)	1 (17.9%)	0.40
1	19 (67.9%)	19 (70.4%)	
≥2	5 (17.9%) (1 missing)	7 (25.9%) (1 missing)	
Uterine size (in cm^3^)			
Mean ± SD	202.7 ± 138.8	266.4 ± 170.9	0.083
Median (range)	184.4 (41.3–543.9)	225.1 (52.0–872.2)	
Amount of menses (in mL)			
Mean ± SD	83.0 ± 10.8	124.2 ± 13.7	9.5 × 10^−11^
Median (range)	84 (63–99.5)	121.5 (111.5–162.5)	
Hemoglobin (in mL)			
Mean ± SD	119.5 ± 16.4	106.9 ± 20.0	0.018
Median (range)	122 (80–140)	111 (66–131)	
Iron supplementation			
No	28 (96.6%)	23 (82.1%)	0.10
Yes	1 (3.4%)	5 (17.9%)	
Severity of dysmenorrhea			
None	3 (10.3%)	3 (10.7%)	0.16
Mild	8 (27.6%)	8 (28.6%)	
Moderate	11(37.9%)	4 (14.3%)	
Severe	7 (24.1%)	13 (46.4%)	
Type of adenomyosis			
Focal	15 (57.6%)	8 (30.8%)	0.093
Diffused	11 (42.4%) (3 unknown)	18 (69.2%) (2 unknown)	
Co‐occurrence with Ovarian endometrioma			
No	17 (58.6%)	22 (78.6%)	0.16
Yes	12 (41.4%)	6 (21.4%)	
Co‐occurrence with Deep endometriosis			
No	17 (58.6%)	22 (78.6%)	0.16
Yes	12 (41.4%)	6 (21.4%)	
Co‐occurrence with Uterine fibroids			
No	14 (48.3%)	17 (60.7%)	0.43
Yes	15 (51.7%)	11 (39.3%)	

Also as expected, we found that women who took iron supplements had significantly greater MBL (*p* = 0.0015) but lower Hb levels (*p* = 7.6 × 10^−5^) than those who did not. While parity was not found to be associated with the amount of MBL (*p *= 0.55), it was negatively associated with the Hb levels (*p *= 0.0088). Patients with diffuse adenomyosis experienced greater MBL and had lower Hb levels, but the difference did not reach statistical significance (*p *= 0.10 and *p *= 0.93, respectively).

### 3.1 Higher lesional stiffness in patients with excessive menstrual bleeding (EXB)

Based on TVESG, we found that lesional stiffness, in terms of LFI, in patients with EXB was significantly higher than that of patients with MHB (*p* = 1.1 × 10^−5^; Figure 3C). Multiple regression analysis incorporating age, parity, menstrual phase, uterine size, co‐occurrence of ovarian endometrioma, deep endometriosis, or uterine fibroids and group identity (MHB vs. EXB) indicated that the group identity was the only co‐variable that was associated with the lesional stiffness, with patients complaining of EXB having significantly higher lesional stiffness than that of participants with MHB (*p* = 3.0 × 10^−6^, *R* ^2^ = 0.38).

While the lesional stiffness did not correlate with the severity of dysmenorrhea (*p *= 0.54), it correlated positively with the amount of reported MBL (*r* = 0.48, *p* = 0.0004; Figure 3D). It appeared to be correlated negatively with the measured Hb levels, but it did not reach statistical significance (*r* = −0.26, *p* = 0.073; Figure 3E). There was no significant difference in lesional stiffness between diffuse and focal adenomyosis (*p* = 0.43) or between proliferative and secretory phases (*p* = 0.27).

Multiple linear regression with MBL as the dependent variable and age, parity, uterine size, type of adenomyosis (focal vs. diffuse), co‐occurrence of ovarian endometrioma, deep endometriosis, or uterine fibroids, use of iron supplements, and lesional stiffness indicated that lesional stiffness, diffuse type of adenomyosis and taking iron supplements were positively associated (*p* = 0.008, *p* = 0.011, and *p* = 0.0001, respectively), while the co‐occurrence of ovarian endometrioma was negatively associated (*p* = 0.045) with MBL (*R^2^* = 0.56). Similarly, uterine size and taking iron supplements were negatively associated (*p* = 0.0008, and *p* = 6.0 × 10^−12^, respectively) with the Hb levels (*R^2^* = 0.65).

The tissue stiffness, in terms of LFI, in eutopic endometrium was similar between participants with MHB and EXB (*p* = 0.14), so too was the stiffness in the EMI tissues (*p* = 0.56). However, the LFI detected in both EMI and endometrial tissues sometimes gave negative values, an indication that the built‐in regression that led to the LFI is ill‐suited to these two cases. Consistent with the increased lesional LFI, the difference in inverted strain ratio of lesions vs. eutopic endometrium between the two groups did not reach statistical significance (*p* = 0.084; Figure 3F).

### 3.2 Evidence for reduced COX‐2 signaling in adenomyotic lesions

**FIGURE 4:**
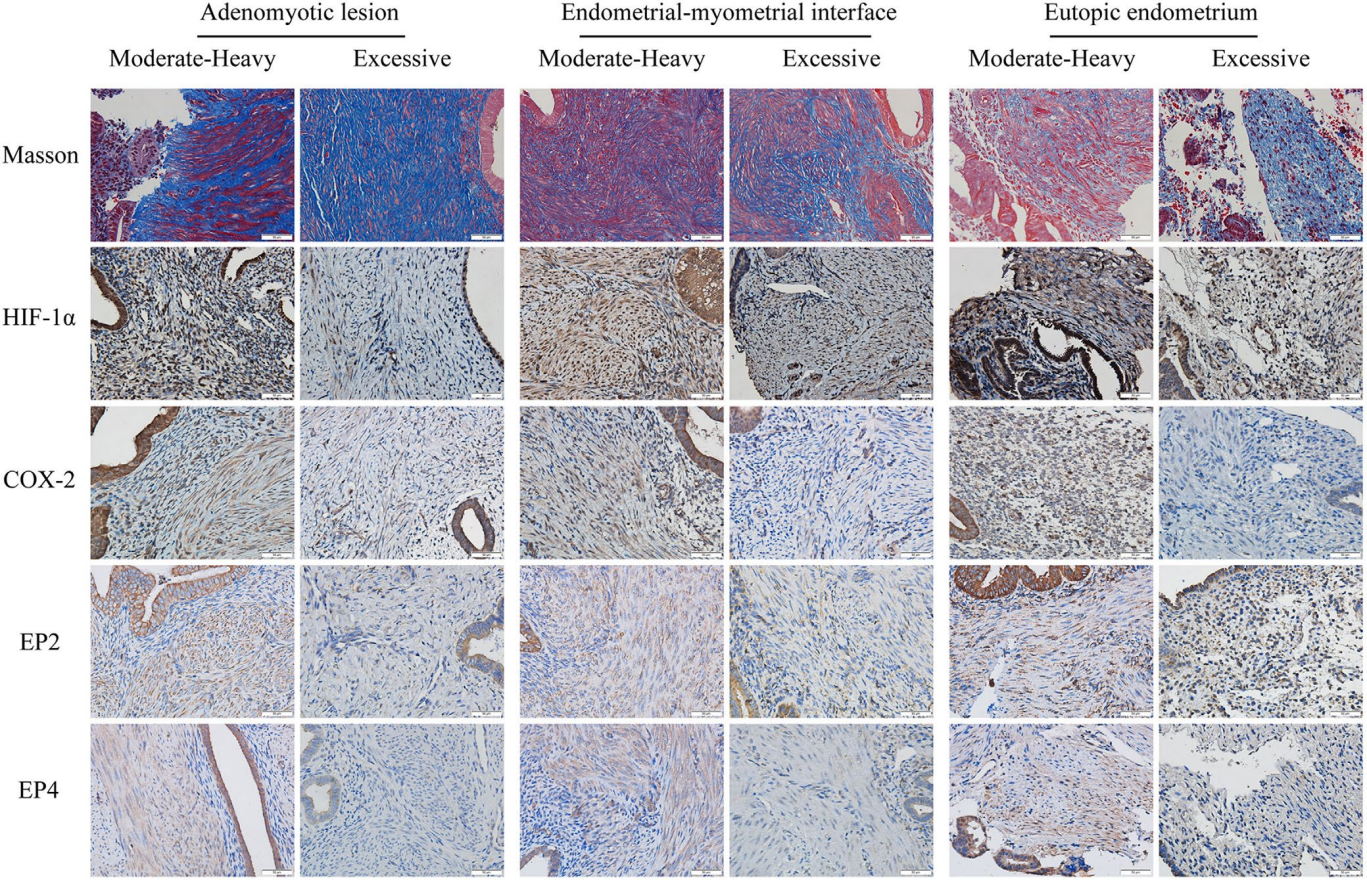
Representative photomicrographs of immunohistochemistry and histochemistry (Masson trichrome) analyses of HIF‐1α, COX‐2, EP2, and EP4, along with the extent of fibrosis in adenomyotic lesions (left panel), their neighboring endometrial–myometrial interface or EMI (middle panel) and eutopic endometrium (right panel) in patients with adenomyosis complaining of moderate–heavy MBL (MHB) and excessive MBL (EXB). The definitions for MHB and EXB are given in the text and Figure 1. Collagen fibers were stained blue, and muscle fibers were red with Masson trichrome staining. Immunoreactivity of HIF‐1α, COX‐2, EP2, and EP4 was observed in both epithelial cells and stromal cells, and HIF‐1α localized both in the cytoplasm and nucleus, while COX‐2 localized in the cell cytoplasm, and EP2 and EP4 in the cell membrane. Magnification: ×400. Scale bar = 50 μm. HIF‐1α indicates hypoxia inducing factor 1α; COX‐2, cyclooxygenase‐2; EP2 and EP4, E‐series of prostaglandin receptors Type 2 and Type 4

Having established the first link between degree of MBL and the extent of lesional stiffness, but not of the stiffness of the neighboring EMI and eutopic endometrium due possibly to the peculiarity of the strain elastography, we next evaluated and compared the fibrotic content in adenomyotic lesions and their neighboring EMI and endometrium between patients with MHB and EXB. In addition, we performed IHC analyses of HIF‐1α, COX‐2, EP2, and EP4 for adenomyotic lesions, as well as EMI and eutopic endometrial tissue samples. We found that the staining of HIF‐1α, COX‐2, EP2, and EP4 was seen in both epithelial and stromal cells, and HIF‐1αstaining was localized both in the cytoplasm and nucleus, while COX‐2 staining was localized in the cell cytoplasm, and EP2 and EP4 in the cell membrane (Figure 4; Figure S2).

**FIGURE 5:**
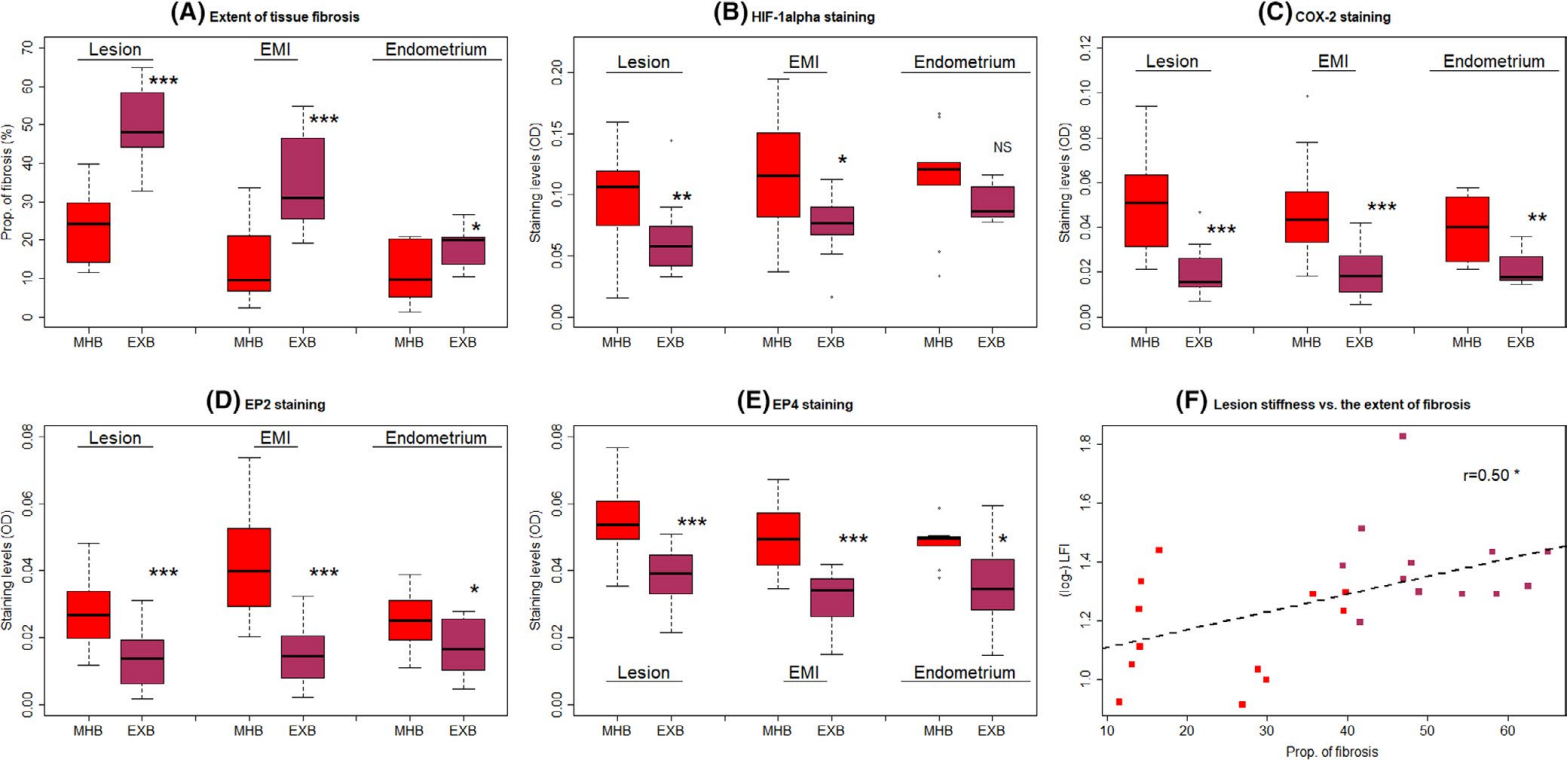
Boxplots showing summary results of histochemistry (Masson trichrome staining) and immunohistochemistry of HIF‐1α, COX‐2, EP2, and EP4 in different tissues (adenomyotic lesions, their neighboring endometrial–myometrial interface (EMI) and eutopic endometrium) from patients with adenomyosis complaining of moderate–heavy MBL (MHB) and excessive MBL (EXB). The definitions for MHB and EXB are given in the text. (A) Extent of tissue fibrosis, as evaluated by Masson trichrome staining. The staining levels of HIF‐1α (B), COX‐2 (C), EP2 (D), and EP4 (E) in the two groups of patients. In boxplots, the upper and lower sides of the box represents the upper and lower quartiles, respectively, while the two whiskers represent the minimal and maximal values. The data points outside of the whiskers are outliers. The definitions for MHB and EXB are given in the text and Figure 1. (F) Scatter plot showing the relationship between the lesional stiffness (LFI) as measured by elastosonography and the extent of lesional fibrosis as evaluated by Masson trichrome staining. The dashed line represents the regression line. The Pearson's correlation coefficient is shown in the figure, along with its statistical significance level. Symbols for statistical significance levels: #: 0.05 < *p *< 0.08; *: *p *< 0.05; **: *p* < 0.01; ***: *p* < 0.001; NS: *p *> 0.10. HIF‐1α, hypoxia‐inducing factor 1α; COX‐2, cyclooxygenase‐2; EP2 and EP4, E‐series of prostaglandin receptors Type 2 and Type 4, respectively

We found that the extent of fibrosis in adenomyotic lesions was significantly higher in the patients with EXB than those with MHB (*p* = 6.3 × 10^−8^; Figure 5A). In addition, adenomyotic lesions from the patients with EXB displayed significantly lower immunostaining levels of HIF‐1α, COX‐2, EP2, and EP4 (*p* = 0.0085, *p* = 8.9 × 10^−6^, *p* = 3.9 × 10^−4^, *p* = 1.5 × 10^−5^; Figure 5B‐E). Multiple linear regression analyses incorporating age, parity, menstrual phase, uterine size, co‐occurrence of ovarian endometrioma, deep endometriosis, or uterine fibroids, and use of iron supplements confirmed that adenomyotic lesions from the patients with EXB displayed significantly higher fibrotic content but significantly lower immunostaining levels of HIF‐1α, COX‐2, EP2, and EP4 (all *p*‐values <0.0083; all *R* ^2^'s ≥ 0.36). Regression analyses also identified uterine size as the co‐variable negatively associated with the COX‐2 staining levels (*p* = 0.030), while being in the secretory phase was positively associated with EP2 staining levels (*p* = 0.025).

The extent of lesional fibrosis correlated negatively with the immunostaining levels of HIF‐1α, COX‐2, EP2, and EP4 (all *r*'s< −0.48, all *p*‐values <0.0051). The immunoreactivity of HIF‐1α and COX‐2 was positively correlated (*r* = 0.57, *p* = 0.007), and that of EP2 and EP4 was also positively correlated (*r* = 0.48, *p* = 0.005). The COX‐2 staining levels were positively correlated with that of EP2 (*r* = 0.39, *p* = 0.027) and of EP4 (*r* = 0.63, *p* = 0.0001). Multiple linear regression analyses incorporating age, parity, uterine size, use of iron supplements or not, type of adenomyosis, and co‐occurrence of ovarian endometrioma, deep endometriosis, or uterine fibroids indicated that diffuse adenomyosis, iron supplements, and lesional stiffness were positively associated (all 3 *p*‐values < 0.012), while the co‐occurrence of ovarian endometrioma was negatively associated with (*p* = 0.045) the amount of MBL (*R* ^2^ = 0.56). Consistent with the lesional stiffness data, there was no significant difference in the extent of lesional fibrosis between diffuse and focal adenomyosis (*p* = 0.91). No difference in lesional HIF‐1α, COX‐2, and EP4 staining was found between the two different menstrual phases (all *p*‐values ≥0.20). The lesional EP2 staining levels were significantly lower in the proliferative phase as compared with the secretory phase (*p* = 0.017).

**FIGURE 6:**
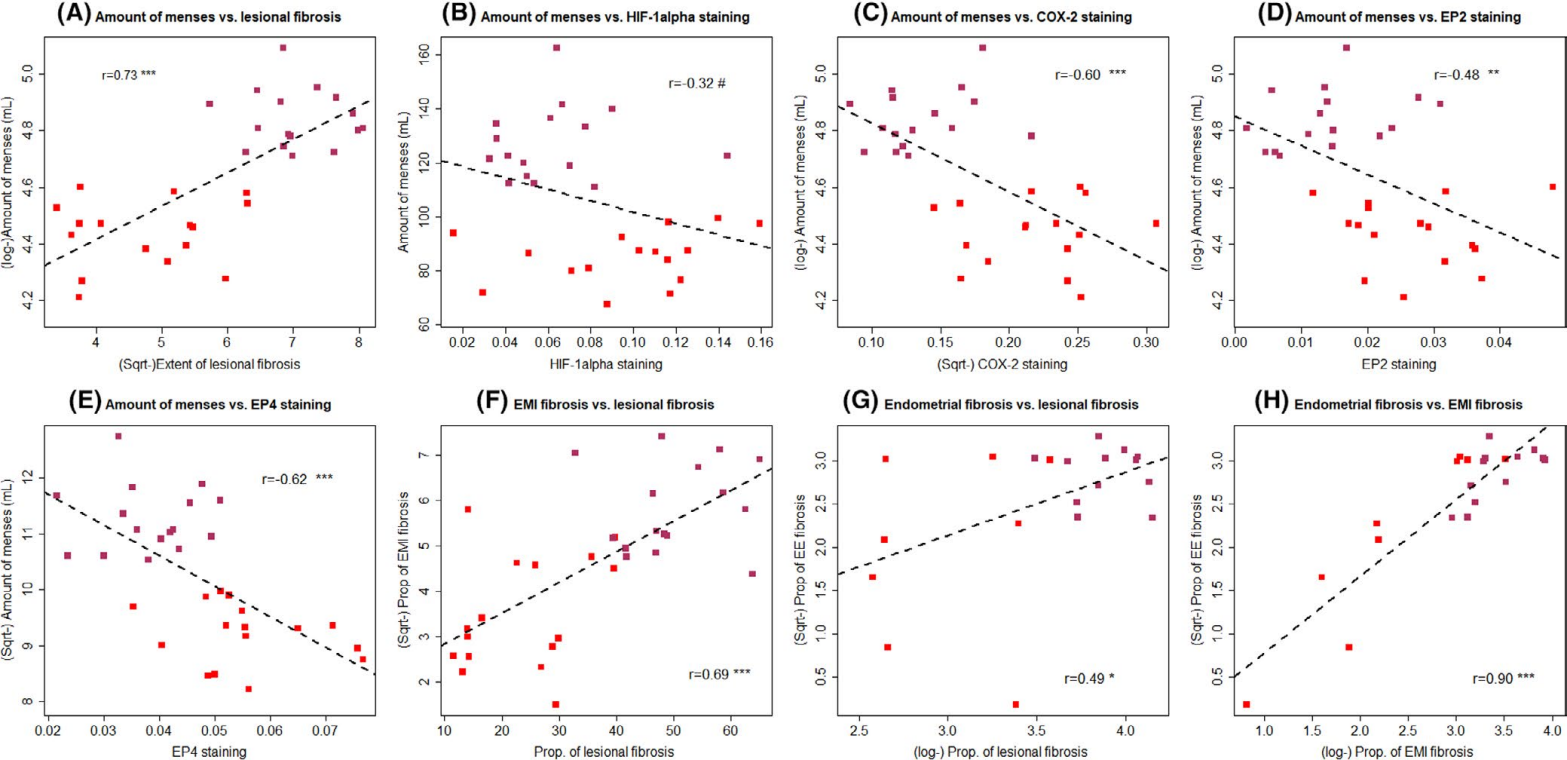
Scatter plot of menstrual blood loss (MBL) versus (A) the extent of lesional fibrosis, (B) HIF‐1α, (C) COX‐2, (D) EP2, (E) EP4 immunostaining levels, (F) the extent of tissue fibrosis in endometrial–myometrial interface (EMI) vs. the extent of lesional fibrosis, (G) the extent of tissue fibrosis in endometrium vs. the extent of lesional fibrosis, and (H) the extent of tissue fibrosis in endometrial–myometrial interface (EMI) vs. the extent of fibrosis in endometrium, in patients with adenomyosis complaining of moderate–heavy MBL (MHB, red‐colored dots) and excessive MBL(EXB, purple‐colored dots). The dashed line represents the regression line. The number shown is the correlation coefficient, along with the symbol showing the levels of statistical significance. The definitions for MHB and EXB are given in the text and Figure 1. Symbols for statistical significance levels: NS: *p *> 0.05; *: *p *< 0.05; **: *p* < 0.01; ***: *p* < 0.001; #: *p* > 0.05. HIF‐1α indicates hypoxia inducing factor 1α; COX‐2, cyclooxygenase‐2; EP2 and EP4, E‐series of prostaglandin receptors Type 2 and Type 4; EMI: endometrial–myometrial interface

Consistent with what we have previously reported,^15^ the lesional stiffness and the extent of lesional fibrosis displayed a positive correlation (*r* = 0.50, *p* = 0.012; Figure 5F). In addition, the amount of MBL correlated positively with the extent of lesional fibrosis (*r* = 0.73, *p* = 1.8 × 10^−6^; Figure 6A). With the exception of HIF‐1α staining levels, the correlation of which with the amount of MBL did not reach statistical significance (*r* = −0.32, *p* = 0.07; Figure 6B), the immunostaining levels of COX‐2, EP2, and EP4 were all negatively correlated with the amount of MBL (*r* = −0.60, *p* = 0.00027, *r* = −0.48, *p* = 0.0051, and *r* = −0.62, *p* = 0.00015; Figure 6C‐E).

The extent of lesional fibrosis correlated with the extent of fibrosis in the neighboring EMI (*r* = 0.69, *p* = 1.1 × 10^−5^; Figure 6F) and in the eutopic endometrium (*r* = 0.49, *p* = 0.023; Figure 6G). Remarkably, the extent of fibrosis in the EMI correlated closely with that in the eutopic endometrium (*r* = 0.90, *p* = 3.6 × 10^−8^; Figure 6H). This is consistent with the notion that fibrosis in lesions can be propagated into their neighboring EMI and eutopic endometrium.

### 3.3 Evidence for reduced COX**‐**2 signaling in the EMI

Similar to the adenomyotic lesions, the extent of EMI tissue fibrosis was significantly higher in the patients with EXB than those patients with MHB (*p* = 5.3 × 10^−6^; Figure 5A). In addition, the EMI tissues from the patients with EXB displayed significantly lower immunostaining levels of HIF‐1α, COX‐2, EP2, and EP4 (*p* = 0.011, *p* = 3.0 × 10^−5^, *p* = 3.2 × 10^−7^, *p* = 1.1 × 10^−5^; Figure 5B‐E). Multiple linear regression analyses incorporating group identity (MHB vs. EXB), age, parity, menstrual phase, uterine size, type of adenomyosis, co‐occurrence of ovarian endometrioma, deep endometriosis, or uterine fibroids and use of iron supplements confirmed that EMI tissues from the patients with EXB had significantly higher fibrotic content but significantly lower immunostaining levels of HIF‐1α, COX‐2, EP2, and EP4 (all *p*‐values<0.0004; all *R^2^* 's ≥ 0.42). Regression analyses also identified that diffuse adenomyosis is *positively* associated with the HIF‐1α staining levels (*p* = 0.0064; *R^2^* = 0.42), while co‐occurrence of ovarian endometrioma was negatively associated with EP2 staining levels (*p* = 0.016; *R^2^* = 0.67). The extent of fibrosis in adenomyotic lesions and the EMI tissues was positively correlated (*r* = 0.69, *p* = 1.1 × 10^−5^), and the 4 immunostaining markers were also positively correlated between adenomyotic lesions and EMI tissues (all *r*'s ≥0.58, all *p*‐values <0.0005).

For EMI tissues, the amount of MBL was positively correlated with the extent of tissue fibrosis (*r* = 0.66, *p* = 3.6 × 10^−5^), but negatively with immunostaining levels of HIF‐1α, COX‐2, EP2, and EP4 (all *r*'s ≤ −0.45, and all *p*‐values <0.0096). No significant difference in staining levels of HIF‐1α, COX‐2, EP2, and EP4 was found between proliferative and secretory phases (all *p*‐values >0.08).

### 3.4 Evidence for reduced COX**‐**2 signaling in eutopic endometrium

We were able to procure 9 and 12 endometrial tissue samples from the full‐thickness tissue blocks of MHB and EXB groups, respectively. Similar to the adenomyotic lesions and the EMI tissues, the extent of tissue fibrosis in eutopic endometrium was significantly higher in patients with EXB than those with MHB (*p* = 0.033; Figure 5A). However, while the eutopic endometrium from the patients with EXB seemed to have lower immunostaining levels of HIF‐1α than MHB patients, the difference did not reach statistical significance (*p* = 0.082; Figure 5B), likely due to the small sample size. The staining levels of COX‐2, EP2, and EP4 in patients with EXB were significantly lower than those with MHB (*p* = 0.0046, *p* = 0.029, *p* = 0.012; Figure 5C‐E). With the only exception for HIF‐1α (*p *> 0.10), in which there was no significant difference between the two groups of patients, multiple linear regression analyses incorporating group identity (MHB vs. EXB), age, parity, menstrual phase, uterine size, type of adenomyosis, co‐occurrence of ovarian endometrioma, deep endometriosis, or uterine fibroids and use of iron supplements confirmed that endometrial tissues from the patients with EXB had significantly higher fibrotic content but significantly lower immunostaining levels of COX‐2, EP2, and EP4 (all *p*‐values ≤0.041; all *R^2^* ≥ 0.22). No significant difference in staining levels of HIF‐1α, COX‐2, EP2, and EP4 was found between proliferative and secretory phases (all *p*‐values >0.30).

The extent of tissue fibrosis in eutopic endometrium correlated positively with that of adenomyotic lesions and the EMI tissues (*r* = 0.49, *p* = 0.023, and *r* = 0.89, *p* = 5.5 × 10^−8^). With the only exception of EP4 staining in adenomyotic lesions (*r* = 0.35, *p* = 0.12), the 4 immunostaining markers in eutopic endometrium were also positively correlated with their counterparts in lesions and the EMI tissues (all *r*'s ≥0.58, all *p*‐values <0.006). In addition, the amount of MBL correlated positively with the extent of tissue fibrosis (*r* = 0.44, *p* = 0.047) but negatively with the COX‐2 staining levels in eutopic endometrium (*r* = −0.49, *p* = 0.028).

### 3.5 Evidence for the gradient of tissue fibrosis

We plotted the extent of tissue fibrosis, and immunostaining levels of HIF‐1α, COX‐2, EP2, and EP4 for all patients across adenomyotic lesions and their neighboring EMI and endometrium (Figure S3). From Figure 5A and Figure S3, it can be seen that the extent of tissue fibrosis appears to have propagated from adenomyotic lesions to their neighboring EMI and then further to the eutopic endometrium. However, the reduced HIF‐1α staining and the COX‐2/EP2/EP4 staining appeared to remain more or less constant, and exhibited significant difference (with the exception of HIF‐1α and EP2 in endometrium due likely to lack of statistical power) between the MHB and EXB groups (Figure 5 and Figure S3B‐E).

## 4 DISCUSSION

We have demonstrated in this study that, in women with adenomyosis who complained of excessive MBL (EXB), the lesional stiffness, as measured by TVEGS, was significantly higher than those who complained of moderate–heavy MBL (MHB). Consistent with the increased lesional stiffness, the extent of lesional fibrosis and the extent of tissue fibrosis in neighboring EMI and eutopic endometrium were also significantly higher. In addition, in adenomyotic lesions and their neighboring EMI and eutopic endometrial tissues, the immunostaining of HIF‐1α (except for eutopic endometrium), COX‐2, EP2, and EP4 was significantly reduced, concomitant with increased tissue fibrosis. This strongly implicates reduced hypoxia and reduced PGE_2_ production and signaling in adenomyosis lesions, as well as their neighboring EMI and endometrial tissues as lesions become highly fibrotic. There is a close but inverse correlation between the extent of fibrosis and the immunostaining levels of HIF‐1α, COX‐2, EP2, and EP4 in adenomyosis lesions and EMI tissues, and also in eutopic endometrium. This reduced hypoxia and PGE_2_ signaling appears to start (presumably) in the adenomyosis lesions first and propagate to their neighboring EMI and then eutopic endometrium, as suggested by close correlation in fibrotic content in these tissues. Given the recent report that adenomyosis lesions are directly connected with their neighboring endometrial glands,^31^ this propagation could conceivably be conducted through cellular communications. Therefore, there is an apparent gradient in the extent of fibrosis, with the adenomyosis lesion having the highest fibrotic content, followed by EMI and then endometrium (Figure 5A and Figures S2,3), suggesting that the progression of adenomyosis lesions impacted the same signaling pathways in the EMI and eutopic endometrium, resulting in impaired endometrial repair and subsequent HMB as observed in mouse models of adenomyosis and menstruation (Mao et al., in preparation). In light of the well‐documented role of PGE_2_ and hypoxia signaling pathways in endometrial repair and HMB,^18^, ^19^, ^20^ our data strongly suggest that the increased lesional fibrosis may contribute to disturbed PGE_2_ signaling in eutopic endometrium and thus possibly reduced hypoxia necessary for endometrial repair, leading to HMB in women with adenomyosis.

As adenomyosis lesions progress and become more fibrotic and thus stiffer, the HIF‐1α and PGE_2_ signaling in these lesions also becomes progressively suppressed. This is because increased adenomyosis lesional fibrosis creates a microenvironment conducive to further fibrogenesis in neighboring tissues and a stiffening ECM, which, in turn, accentuates the actions of transforming growth factor β1 (TGF‐β1) and promotes myofibroblast activation.^43^, ^44^, ^45^ Since adenomyosis lesions are physically connected with endometrial glands,^31^ lesional fibrosis would propagate, perhaps through paracrine actions, to neighboring EMI and then eutopic endometrium, stiffening these tissues. As matrix stiffness increases in lung fibroblasts and interferes with multiple steps of PGE_2_ synthesis, including the suppression of prostaglandin E synthases (PTGESs) that specifically convert precursor PGH_2_ to PGE_2_,^46^ the PGE_2_ production and signaling would subside.^27^, ^47^ The increased fibrosis may also enhance PGE_2_ degradation, as seen in increased expression of the PGE_2_‐degrading enzyme 15‐PGDH in the fibrotic lung.^48^ As a result, there may be a subsequent suppression of PGE_2_ as well as HIF‐1α signaling in adenomyosis lesions and their neighboring EMI and endometrium, with ensuing impaired and disrupted endometrial repair and, ultimately, HMB. Indeed, endometrial stromal cells cultured on a stiff matrix display reduced expression of COX‐2, EP2, and EP4 with or without TGF‐β1 stimulation.^28^ Furthermore, the combined use of a recently established mouse model of adenomyosis^25^ and a mouse model of simulated menstruation^49^ lends further support for this proposal (Mao et al., in preparation).

**FIGURE 7:**
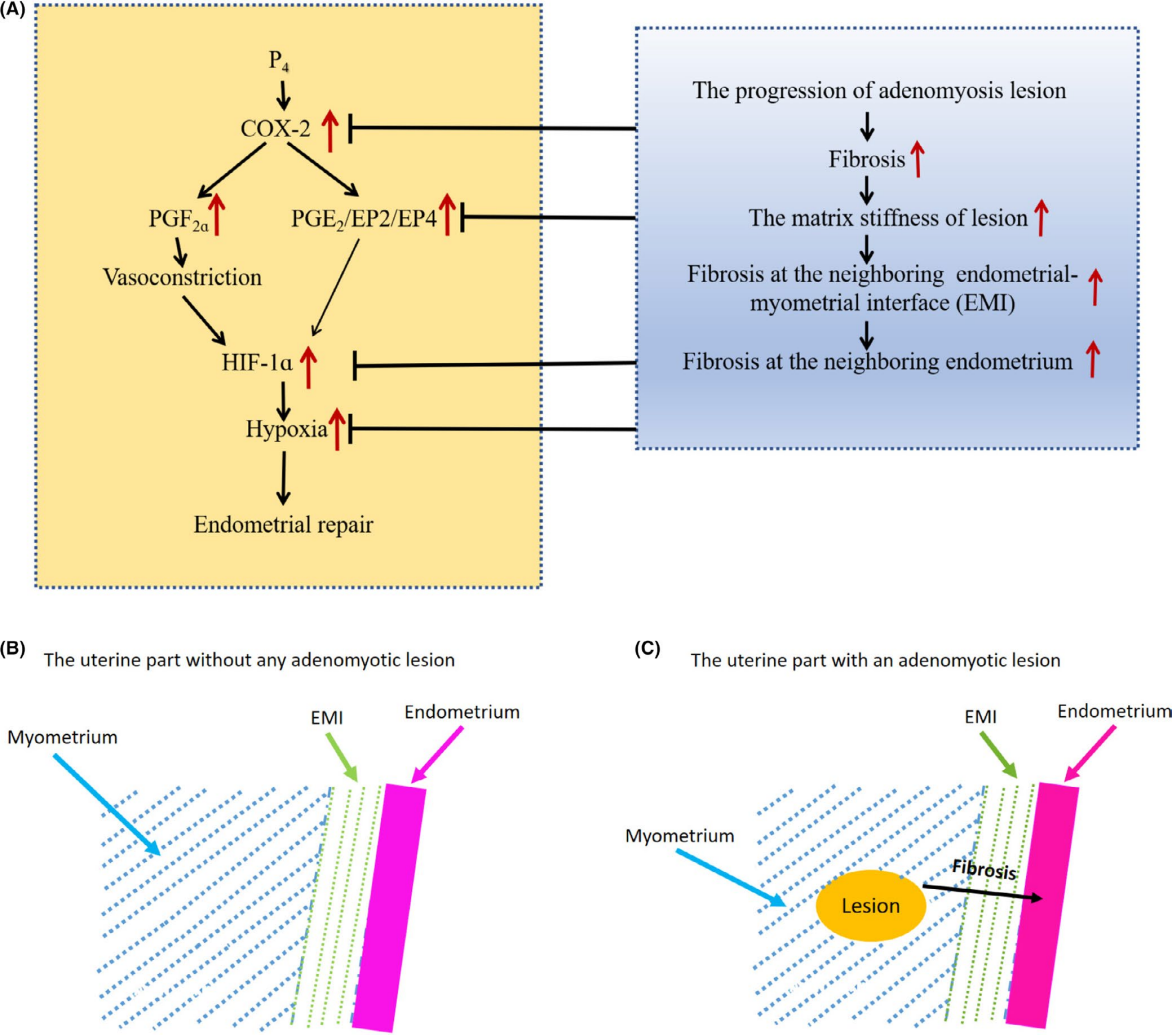
(A) Diagram depicting possible mechanisms of adenomyosis‐associated heavy menstrual bleeding (HMB). The left panel, in yellow background, depicts the known mechanisms underlying heavy menstrual bleeding due to impaired endometrial repair (see review^9^). The right panel, in gray background, sketches the possible mechanisms underlying adenomyosis‐associated HMB. The upward red arrows indicate upregulation/overexpression or increase. The black directional arrows means “lead to” or “result in.” The lines with a tip of a short line indicate “suppression” or “inhibition.” (B) Schematic illustration showing the rather normal endometrial area without any molecular aberration when it is distal to any adenomyotic lesion or when there is no adenomyosis at all. (C) Schematic illustration showing the endometrial area proximal to an adenomyotic lesion, which may harbor certain molecular aberration due to the propagation of lesional fibrosis

As a corollary, adenomyosis lesions closer to endometrium are more likely than those more distant to cause HMB as lesions progress, simply because of the ease of propagation. This seems to be borne out by an early study which reported that shallower penetration of adenomyosis lesions into the myometrium was associated with higher incidence of HMB.^50^ It is also corroborated by a recent report that internal adenomyosis (i.e., lesions proximal to the endometrium) is more likely to be associated with HMB while external adenomyosis (that is distal to the endometrium but proximal to uterine serosa) is more often associated with deep endometriosis (and thus pain).^51^ The possible mechanisms underlying adenomyosis‐associated HMB are depicted in Figure 7A.

Our findings have important clinical implications. First and foremost, given the close relationship between adenomyotic/endometrial stiffness/fibrosis and the amount of MBL, a more objective assessment, by elastography, of the endometrial repair capability may be possible in future. Monitoring treatment responses to therapeutic interventions may also be possible. In addition, one apparent clinical implication would be that the progression of adenomyosis would ultimately impact on menstruation, especially when the lesions are proximal to the endometrium. Hence, given the progressive nature of adenomyotic lesions, early intervention would be the key to the successful management of adenomyosis. Moreover, our finding that endometrial fibrosis propagated from fibrotic adenomyotic lesions may also impact on endometrial receptivity, as reported by Kim et al. in the context of endometriosis.^52^ Lastly, our research may provide new therapeutic targets for future research.

It has not escaped our attention that the presumably causal link between HMB and the suppression of the HIF‐1α and PGE_2_ signaling pathways in endometrium due to increased tissue stiffness resulting from increased lesional fibrosis may also exist for other disorders that cause HMB, such as uterine fibroids and polyps. Remarkably, submucosal uterine fibroids are also associated with HMB,^53^ suggesting that similar mechanisms may also operate in fibroids. This, of course, requires further investigation.

Evidence for reduced HIF‐1α staining, and possibly repressed hypoxia, and PGE_2_ signaling is shown in our present study in adenomyosis lesions and the neighboring EMI and eutopic endometrial tissues collected during either proliferative or secretory phases. Indeed, for endometrial repair, their signaling, or lack thereof, is relevant shortly before and during menstruation in endometrial tissues.^18^, ^20^, ^54^, ^55^ Several lines of evidence suggest that the latter actually happens in eutopic endometrium. First, eutopic endometrium can and does exhibit fibrosis when endometriosis is present.^52^ It is possible that eutopic endometrium from women with adenomyosis may also become fibrotic, as presented herein. Second, fibrosis inevitably begets tissue stiffening, which turns fibroblasts from a quiescent state to progressive increases in proliferation and synthesis of ECM products, concomitant with decreases in matrix proteolytic gene expression, reduced expression of COX‐2 and synthesis of PGE_2_,^27^ as occurred in endometriosis.^28^, ^29^ In fact, PGE_2_ is reported to be anti‐fibrotic.^21^, ^23^, ^56^, ^57^, ^58^ This should take place irrespective of menstrual cycle phase, as found in our regression analyses. Third, it has been reported that, in women with adenomyosis, there is no difference in COX‐2 staining between ectopic and eutopic endometrium.^59^ Hence, the immunostaining levels of COX‐2 and possibly EP2 and EP4 as well are likely to be similar in the two types of tissues, as seen in Figure 5. Lastly, in some fibrotic diseases, EP2 expression is reduced,^58^, ^60^, ^61^ likely due to promoter hypermethylation.^62^ EP4 may also be reduced.^58^

Our data are consistent with our previous report that the adenomyosis lesional stiffness, as measured by LFI on strain elastography, correlated positively with the extent of lesional fibrosis.^15^ These data are also consistent with our reports that the extent of adenomyosis lesional fibrosis correlated with uterine size,^10^, ^15^ as the enlarged uterus is likely the result of progressive EMT, FMT, and SMM.^10^, ^11^ The seemingly reduced lesional staining of HIF‐1α as the extent of adenomyosis lesional fibrosis increased is consistent with the report that deep endometriotic lesions have significantly lower HIF‐1α expression levels than that of ovarian endometriomas,^63^ since the former is well known to be more fibrotic than the latter.^64^

Our data are however in conflict with several reports of elevated endometrial COX‐2^65^, ^66^ and HIF‐1α^67^, ^68^ expression in adenomyosis. Our data may also seem to be at odds with the increased endometrial expression of COX‐2 in women with HMB,^69^ the endometrial HIF‐1α expression restricted only in the peri‐menstrual phase^18^, ^20^, ^70^ as well as the temporal expression patterns of EP2 and EP4.^71^ There are several reasons for these apparent discrepancies, as elaborated below.

First, these data themselves are not always entirely consistent, and aspects of previously published data are congruent with our data. For example, in Ota et al., the COX‐2 staining levels in both eutopic and ectopic endometrium from women with adenomyosis were lower than that of the endometriosis group.^65^ However, it did *not* specifically compare the COX‐2 staining levels between control and adenomyosis groups. In fact, the data presented indicate staining levels between these two groups are comparable.

Second, while the comparison was made between women with and without adenomyosis in these studies, the specific symptoms of the recruited patients are either unstated or simply different from those herein. For example, the women with adenomyosis in Li et al.^66^ were those who complained exclusively of dysmenorrhea, which might have entirely different underlying mechanisms. As for Smith et al.,^69^ while this study reported higher COX‐2 expression in endometrium from women with HMB as compared with controls, the patients with HMB were specifically identified to *exclude* those with adenomyosis or fibroids. In contrast, our study recruited exclusively patients with adenomyosis who complained of HMB, and, as such, their adenomyosis lesions may be intrinsically more fibrotic.

Third, in all these studies, no attempt was made to quantify the extent of lesional fibrosis, or the extent of tissue fibrosis in the EMI and eutopic endometrium. An important component in our hypothesis is that ECM stiffness resulting from tissue fibrosis is of paramount importance in determining impact upon PGE_2_ and hypoxia signaling.

Lastly, and very importantly, there is a crucial difference in the *location* of tissue sampling. In all these previous studies, the locations of tissue samples appear to be chosen at random or based on convenience. In contrast, the endometrial samples collected in our present study were all adjacent to the adenomyosis lesions that had the highest stiffness and fibrotic content (Figure 7C). It has been well documented that endometrium is polyclonal^72^ and that endometrial loss and regeneration are piecemeal processes that occur simultaneously in different areas of the endometrium.^73^ This strongly suggests that the endometrium is patchy and thus *heterogeneous* (rather than a homogenous structure) and that not all areas of endometrium would contribute equally to HMB. Indeed, the fact that adenomyosis lesions are physically connected with endometrial glands,^31^ in conjunction with the multi‐clonal nature of endometrium,^72^, ^74^ lends further support for this heterogeneity. Emerging data from targeted sequencing,^75^ whole‐genome sequencing,^76^ and single‐cell transcriptomic^77^, ^78^ studies of endometrium also corroborate this heterogeneity. This would be consistent with fibrosis in adenomyosis lesions facilitating propagation to the endometrium and that the endometrium proximal to the adenomyosis lesions may be genuinely different from those distal to the lesions.

Our study has several strengths. First, our hypothesis was anchored on several known biological facts, that is, PGE_2_‐hypoxia signaling in endometrial repair, the progressive fibrogenesis in adenomyosis lesions due to ReTIAR, the close relationship between fibrosis and tissue stiffness, and the positive feedback loop between extracellular matrix stiffness and PGE_2_ production. Second, by the combined use of elastography to detect tissue stiffness and histochemical/ immunohistochemical analyses of full‐thickness uterine tissues (myometrial and endometrial tissues), we were able to establish a chain of evidence linking adenomyosis lesional stiffness with HMB. Lastly, we harvested selected uterine tissue columns harboring the stiffest lesion and the neighboring EMI and endometrial tissues. These findings, accompanied by ultrasound elastography, raise the possibility for the application of a routine, non‐invasive elastographic examination to assist in the clinical assessment of patients with adenomyosis and symptom of HMB, choice of the best treatment modality, and in monitoring patient responses to treatment.

Our study also has several limitations. First, we only compared women with adenomyosis from two groups, MHB and EXB. Ideally, our study would have been enhanced if we also included women with adenomyosis with mild menstrual bleeding, as well as women without adenomyosis/endometriosis/fibroids. Recruitment of the latter group is challenging as they do not present to clinical services. Alternatively, in addition to the ipsilateral uterine tissue columns containing adenomyotic lesions, the contralateral uterine tissue samples in focal adenomyosis could have been used, and this would have provided an unequivocal evidence for endometrial heterogeneity as far as HMB is concerned. Regardless, our data clearly show that the positive correlation of the amount of MBL with the extent of adenomyosis lesional fibrosis, but negative correlation with lesional staining of HIF‐1α, COX‐2, EP2, and EP4 (Figure 5), suggesting that this correlation is likely to hold also for women with adenomyosis with light menses. Second, for study of PGE_2_ signaling, we only evaluated the immunostaining of COX‐2, EP2 and EP4, but not at transcriptional and translational levels. Nor did we evaluate the expression of PTGESs or the degradation of PGE_2_. While increased PGE_2_ degradation and reduced PTGES and thus PGE_2_ production as matrix stiffness increases have been reported in other diseases,^46^, ^48^ future research is warranted to validate this in eutopic endometrium from women with adenomyosis. Third, hypoxia is known to be critical to endometrial repair.^20^ Our study only evaluated the endometrial staining of HIF‐1α, not exactly hypoxia *per se*. While the hallmark of hypoxia is the activation of HIF‐1α,^79^ future studies are needed for confirmation. Lastly, we acknowledge that aside from the hypoxia and PGE_2_ signaling pathways, other mechanisms, such as abnormal vascular maturation and vascularization,^80^, ^81^ coagulation,^82^ and even inherited bleeding disorders,^83^ may also be involved in causing HMB.

In conclusion, we have provided preliminary evidence that the extent of adenomyosis lesional fibrosis, which may be quantified by elastography, correlated positively with the amount of MBL in women with adenomyosis who complained of HMB. In addition, we found that the increased adenomyosis lesional fibrosis is accompanied by reduced HIF‐1α and PGE_2_ immunostaining and possibly signaling in lesions and their neighboring EMI and endometrium, which may result in impaired endometrial repair and subsequent HMB. If further validated, the general underlying mechanism, that is, adenomyosis lesional or tissue fibrosis results in HMB, may be highly relevant in other gynecologic conditions, such as uterine fibroids and polyps. This, coupled with the rising popularity of and further advancement in elastography, should further assist the clinical assessment and also enhance our understanding of underpinning mechanisms involved in patients with the symptom of HMB, thereby paving the way for the development of novel therapeutics.

## CONFLICT OF INTEREST

H.C. has received clinical research support for laboratory consumables and staff from Bayer AG and provides consultancy advice (but with no personal remuneration) for Bayer AG, PregLem SA, Gedeon Richter, Vifor Pharma UK Ltd, AbbVie Inc.; Myovant Sciences GmbH. H.C. receives royalties from UpToDate for article on abnormal uterine bleeding. S.‐W.G. provides consultancy advice for MSD R&D, Chugai Pharmaceutical Co., and BioHaven Pharmaceuticals. All other authors have no conflicts to declare.

## HUMAN RIGHTS STATEMENTS AND INFORMED CONSENT

All procedures followed were in accordance with the ethical standards of the responsible committee on human experimentation (institutional and national) and with the Helsinki Declaration of 1964 and its later amendments. Informed consent was obtained from all patients for being included in the study. Each patient enrolled in this study signed an informed consent for all the procedures and to allow data collection and analysis for research purposes.

## APPROVAL BY ETHICS COMMITTEE

The study was approved by the institutional ethics review board of Shanghai OB/GYN Hospital, Fudan University (March 6, 2019; on file).

## Supporting information

Supplementary Material

Fig S1

Fig S2

Fig S3
