# Supplementary material for: How does the extent of fibrosis in adenomyosis lesions contribute to heavy menstrual bleeding?
Source: Reprod Med Biol. 2022 Feb 7;21(1):e12442. doi: 10.1002/rmb2.12442 (PMC8967287; doi:10.1002/rmb2.12442)
Supplement: Supplementary file 1 — Supplementary Material [file RMB2-21-e12442-s002.docx]

**How does the extent of fibrosis in adenomyosis lesions contribute to**

**heavy menstrual bleeding?**

**Qingqing Huang, Xishi Liu, Hilary Critchley, Zhongpeng Fu, Sun-Wei Guo**

**Supplementary Information**

**Immunohistochemical (IHC) analyses**

The positive staining was evaluated using a semi-quantitative scoring system, as previously described (1, 2). Briefly, the number and intensity of positive cells were counted by Image-Pro Plus 6.0 (Media Cybernetics Inc, Bethesda, MA, USA). A series of 3 to 5 randomly selected images on several sections were taken to obtain a mean value. IHC parameters assessed in the area detected included (a) integrated optical density (IOD); (b) total stained area (S); and (c) mean optical density (MOD), which is defined as MOD= IOD/S, equivalent to the intensity of stain in all positive cells.

The primary antibody employed against HIF-1α, COX-2 and EP4 were all rabbit polyclonal antibodies, and EP2, a rabbit monoclonal antibody. Therefore, for negative controls, human adenomyotic tissue samples were incubated with rabbit serum instead of primary antibodies. For positive controls, human adenomyotic tissues were used for HIF-1α (3), human colorectal tumor tissues for COX-2, human colon tissues for EP2 and lung cancer tissues for EP4. Representative pictures of positive and negative controls are displayed in Supplementary Figure S1.

**Masson trichrome staining**

Masson trichrome staining was employed to detect the extent of collagen fibers in full-thickness uterine tissue samples, including uterine adenomyotic lesions, endometrial-myometrial interface (EMI) and eutopic endometrium. After tissue slides were incubated at 60℃ for 1hour, routine deparaffinization and rehydration procedures were performed in xylene and a graded series of ethanol respectively. Slides were then immersed in Bouin’s solution at 37°C for 2 hours, which is a mixture of saturated picric acid 75 mL, 10% (w/v) formalin solution 25 mL and acetic acid 5 mL. Sections were stained using the Masson’s Trichrome Staining kit (Servicebio, Wuhan, China) following the manufacturer’s instructions. Use of Image Pro-Plus 6.0 permitted the calculation of the blue-stained areas of the collagen fiber layer in proportion to the entire field of uterine adenomyotic lesions, EMI or eutopic endometrium.

**References**

1. Ding D, Liu X, Duan J, Guo SW. Platelets are an unindicted culprit in the development of endometriosis: clinical and experimental evidence. Hum Reprod 2015;30:812-32.

2. Zhang Q, Duan J, Olson M, Fazleabas A, Guo SW. Cellular changes consistent with epithelial-mesenchymal transition and fibroblast-to-myofibroblast transdifferentiation in the progression of experimental endometriosis in baboons. Reprod Sci 2016;In press.

3. Critchley HO, Osei J, Henderson TA, Boswell L, Sales KJ, Jabbour HN *et al.* Hypoxia-inducible factor-1alpha expression in human endometrium and its regulation by prostaglandin E-series prostanoid receptor 2 (EP2). Endocrinology 2006;147:744-53.

**Figure S1. Positive and negative controls for immunohistochemistry.** Representative immunostaining of HIF-1α, COX-2, EP2 and EP4 in human adenomyotic tissue (HIF-1α), human colorectal tumor tissue (COX-2), human colon tissue (EP2) and human lung cancer tissue (EP4), (Left column) shown as positive controls. The negative controls were stained in human adenomyotic tissue samples (Right column). Magnification in all figures: X400. The scale bar represents 50 μm.

**Figure S2. A digitally amplified version of Figure 4.** Representative photomicrographs of immunohistochemistry and histochemistry (Masson trichrome) analyses of HIF-1α, COX-2, EP2 and EP4, along with the extent of fibrosis in adenomyotic lesions (left panel), their neighboring endometrial-myometrial interface or EMI (middle panel) and eutopic endometrium (right panel) in patients with adenomyosis complaining of moderate-heavy MBL (MHB) and excessive MBL (EXB). The definitions for MHB and EXB are given in the text and Figure 1. Collagen fibers were stained blue and muscle fibers were red with Masson trichrome staining. HIF-1α, COX-2, EP2 and EP4 immunoreactivity was observed in both epithelial cells and stromal cells, and HIF-1α localized both in the cytoplasm and nucleus, while COX-2 localized in the cell cytoplasm, and EP2 and EP4 in the cell membrane. The slides used were the same to Figure 4 but were amplified digitally on computer using Photoshop (version CC 20.4, Adobe Systems, San Jose, CA, USA). Magnification: ×1200. Scale bar = 16.76 μm. HIF-1α indicates hypoxia inducing factor 1α; COX-2, cyclooxygenase-2; EP2 and EP4, E-series of prostaglandin receptors Type 2 and Type 4; EMI, endometrial-myometrial interface.

**Figure S3.** The extent of tissue fibrosis (A), immunostaining of HIF-1ɑ (B), COX-2 (C), EP2 (D), and EP4 (E) across lesions, their neighboring EMI and endometrium of the full-thickness uterine tissue samples taken at the site where the adenomyotic lesions had the highest stiffness reading by TVESG or palpation. Each line represents one data point from one patient, with red and maroon lines designating patients from the MHB and EXB groups, respectively. The diamonds indicate the median values. EMI: endometrial-myometrial interface.
