# Supplementary figures and images for: How does the extent of fibrosis in adenomyosis lesions contribute to heavy menstrual bleeding?
Source: Reprod Med Biol. 2022 Feb 7;21(1):e12442. doi: 10.1002/rmb2.12442 (PMC8967287; doi:10.1002/rmb2.12442)

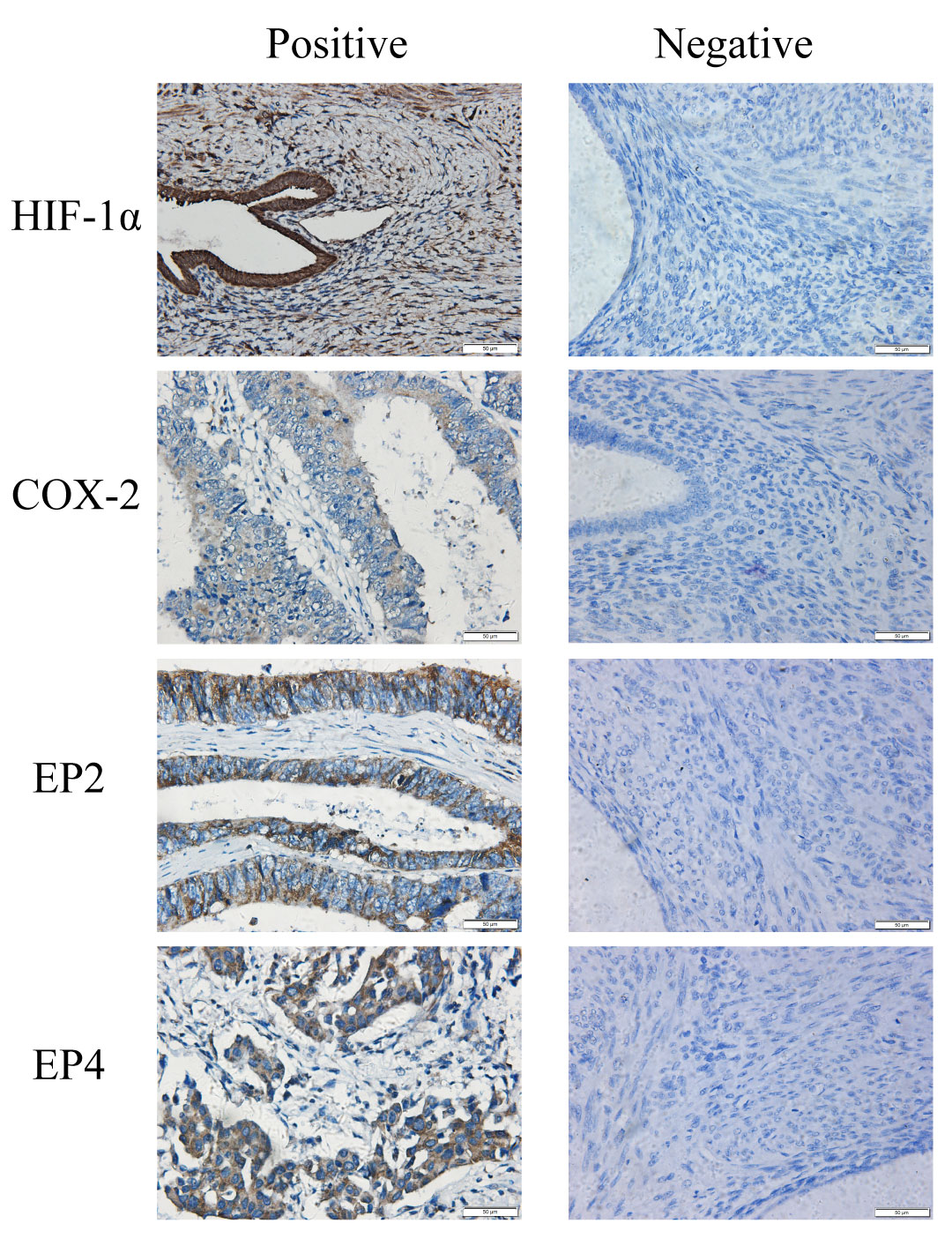

Supplement: Supplementary file 2 — Fig S1 [file RMB2-21-e12442-s003.jpg]

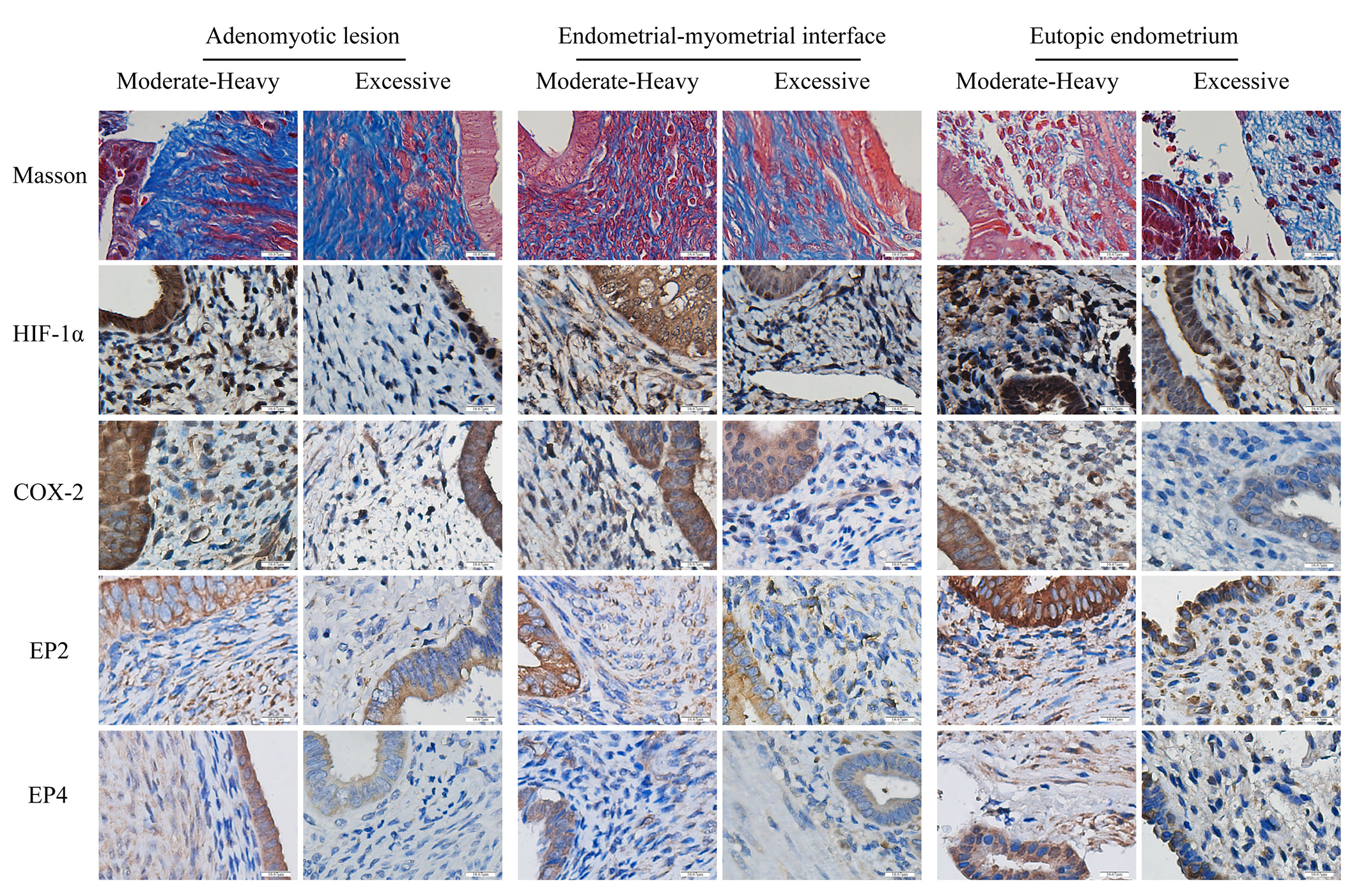

Supplement: Supplementary file 3 — Fig S2 [file RMB2-21-e12442-s001.jpg]

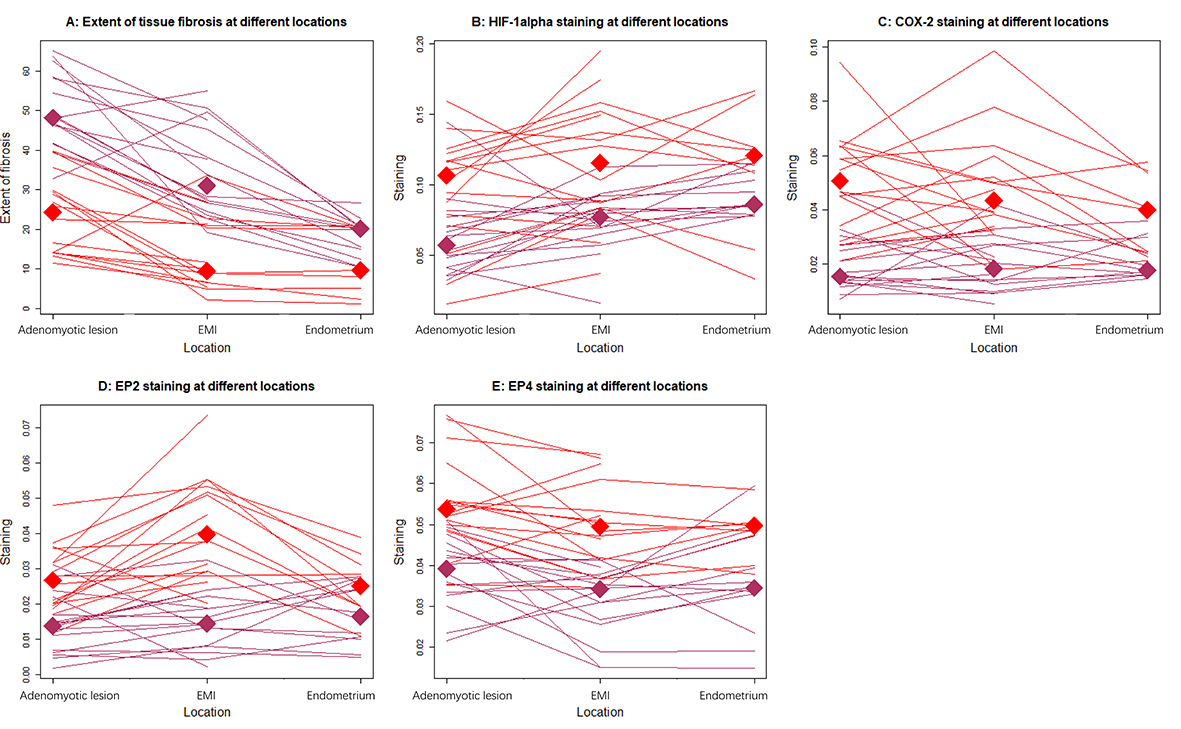

Supplement: Supplementary file 4 — Fig S3 [file RMB2-21-e12442-s004.jpg]
